# TEMPUS, a Timepix4-based system for the event-based detection of X-rays

**DOI:** 10.1107/S1600577524005319

**Published:** 2024-07-23

**Authors:** Jonathan Correa, Alexandr Ignatenko, David Pennicard, Sabine Lange, Sergei Fridman, Sebastian Karl, Leon Lohse, Björn Senfftleben, Ilya Sergeev, Sven Velten, Deepak Prajapat, Lars Bocklage, Hubertus Bromberger, Andrey Samartsev, Aleksandr Chumakov, Rudolf Rüffer, Joachim von Zanthier, Ralf Röhlsberger, Heinz Graafsma

**Affiliations:** a Deutsches Elektronen-Synchrotron DESY Notkestrasse 85 22607Hamburg Germany; b Center for Free-Electron Laser Science – CFEL Deutsches Elektronen-Synchrotron DESY Notkestrasse 85 22607Hamburg Germany; c Friedrich Schiller University Jena, 07743Jena, Germany; d University of Erlangen-Nuremberg, Schlossplatz 4, 91054Erlangen, Germany; e Germany European XFEL GmbH Holzkoppel 4 22869Schenefeld Germany; f European Synchrotron Radiation Facility, 71 Avenue des Martyrs, 38000Grenoble, France; g Helmholtz-Institut Jena, 07743Jena, Germany; h GSI Helmholtzzentrum für Schwerionenforschung GmbH, 64291Darmstadt, Germany; RIKEN SPring-8 Center, Japan

**Keywords:** X-ray detector, photon science, event-driven, sparse-readout, Timepix4

## Abstract

TEMPUS is a new detector system being developed for photon science. It is based on the Timepix4 chip and, thus, it can be operated in two distinct modes: a photon-counting mode, which allows for conventional full-frame readout at rates up to 40 kfps; and an event-driven time-stamping mode, which allows excellent time resolution in the nanosecond regime in measurements with moderate X-ray flux. In this paper, the initial prototype, a single-chip device, is introduced, and the readout system described. Moreover, and in order to evaluate its capabilities, some tests were performed at PETRA III and ESRF for which results are also presented.

## Abstract 2

A full description of the TEMPUS system for photon science is given. The detector takes advantage of the new Timepix4 readout chip and, in particular, implements the use of the time-stamping mode for high-resolution timing applications.

## 1. Introduction

Deutsches Elektronen-Synchrotron (DESY) in Hamburg, Germany, operates one of the most intense storage ring light sources (SR) in the world: PETRA III (Franz *et al.*, 2006 ▸). The upcoming upgrade of the facility to a fourth-generation SR, the PETRA IV project (Schroer *et al.*, 2018 ▸), will increase the brilliance by orders of magnitude, enabling for example highly time-resolved measurements and rapid high-resolution scanning of macroscopic samples. In order to take full advantage of its elevated brilliance, and to record as many photons as possible with their relevant properties like energy, momentum, arrival time *etc.*, DESY is developing fast and efficient X-ray detectors (Graafsma *et al.*, 2023 ▸). Photon-counting detectors are widely used at synchrotron beamlines, due to their sensitivity to single photons and high dynamic range. The LAMBDA detector system (Pennicard *et al.*, 2011 ▸), a photon-counting detector based on the Medipix3 chip (Ballabriga *et al.*, 2007 ▸), has become the detector of choice for a variety of beamlines. The main reason for this is the combination of small pixel size, 55 µm, and a relatively high frame rate, up to 2 kfps (kilo frames per second) in 12 bit depth counter in single threshold mode. A new readout chip has been recently produced by the Medipix4 collaboration – Timepix4 (Llopart *et al.*, 2022 ▸) – which goes beyond the photon-counting concept by extracting more information from each photon. On the one hand, it can work in a full-frame readout mode with much higher count rate and frame rate than Medipix3. On the other hand, it can work in the time-stamping mode improving significantly over the Timepix3 chip (Poikela *et al.*, 2014 ▸).

The Timepix4-based Edgeless Multi-PUrpose Sensor (TEMPUS) detector is being developed as a replacement for LAMBDA. In addition, as will be discussed later, the timing capabilities of the new chip open the door to other applications such as nuclear resonance scattering (NRS) techniques, where time resolutions below the nanosecond regime are desired, and X-ray photon correlation spectroscopy (XPCS) on (sub-)microsecond time scales. In this paper, the system in its current prototype state is described in Section 2. The results of the very first experiments at PETRA III and ESRF, together with some preliminary calibration, are reported in Section 3. Finally, in Section 4 the project plans for the short-term are discussed.

## 2. System description

In order to boost the development speed in this first stage, and bring the system to the diverse user communities as soon as possible, a number of decisions were made during the design phase, which are discussed here.

**Figure 1:**
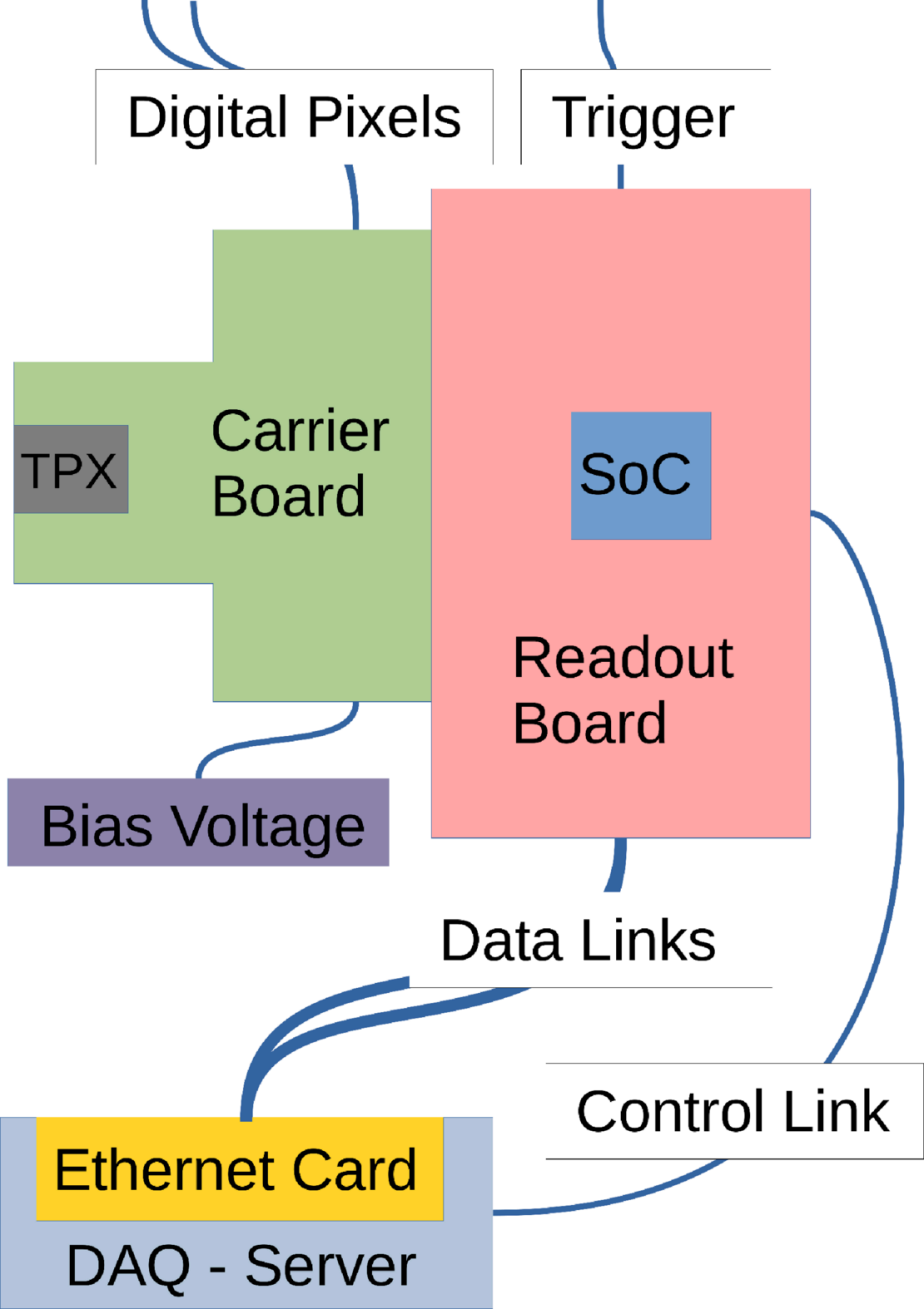
Operation scheme of the TEMPUS single-chip prototype. Besides the readout and carrier boards, the different inputs (triggers, *etc*.) and also the control and data links are shown.

**Figure 2:**
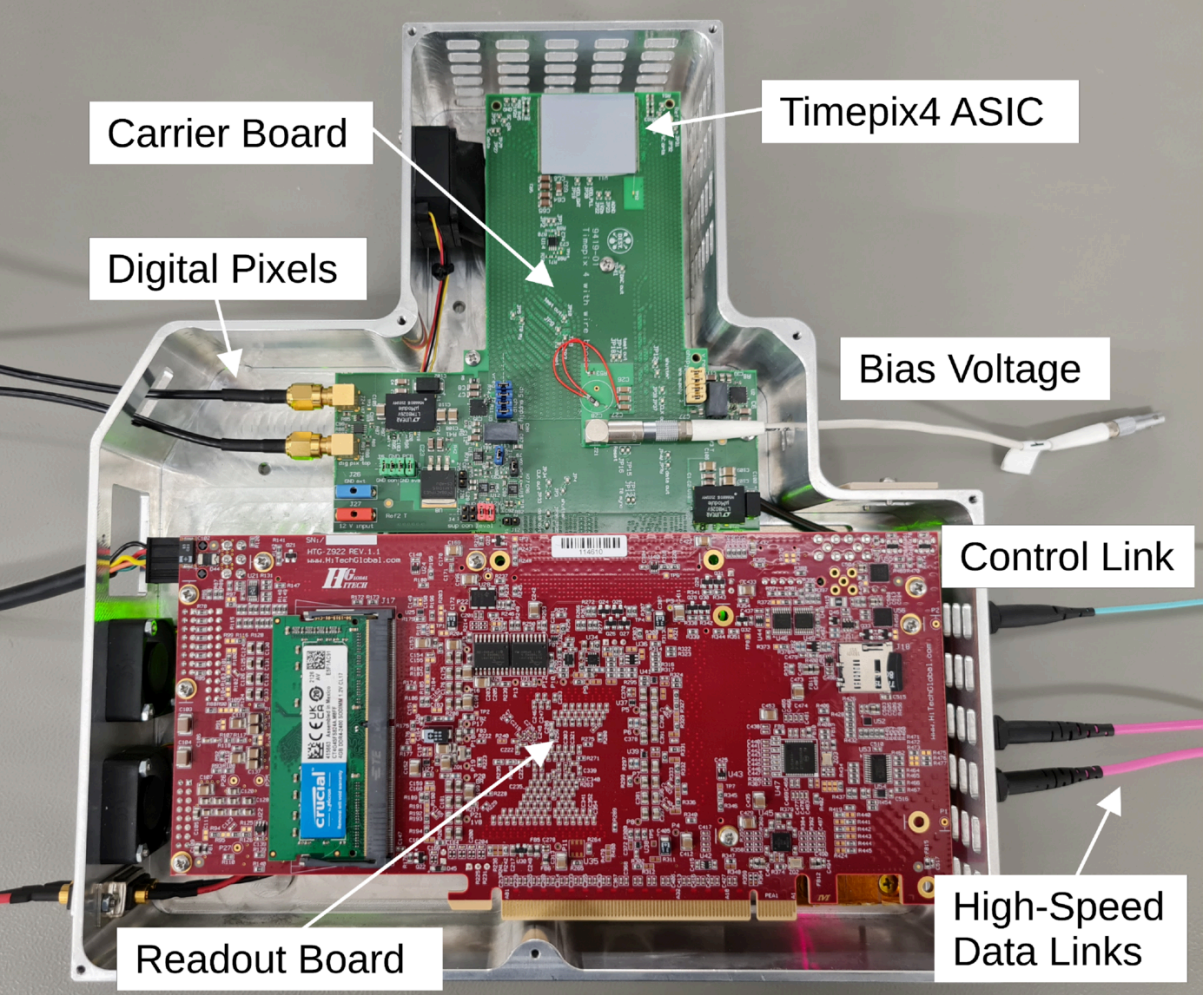
The TEMPUS single-chip prototype in its housing. The design of the carrier board allows for minimal dead area when a second system is placed on the top. Inputs to the system such as bias voltage, external trigger or digital pixels can be seen. Also, the optical cables connecting to the slow control and the high-speed data-links are visible.

First and foremost, the larger area of the Timepix4 chip (694 mm^2^) compared with previous generations (3.5 times larger than Medipix3) allows us to cover a sufficiently large solid angle without mounting several chips in this first prototype. We therefore opted for a single-chip approach. Also, little optimization was made in terms of form factor in the design of the chip carrier board. Finally, we opted to use a powerful and commercially available Xilinx system-on-chip (SoC) evaluation board for readout. All these different components will now be individually described in this section. A full scheme of the system is shown in Fig. 1 ▸, and a photograph of the TEMPUS single-chip readout prototype is shown in Fig. 2 ▸.

### 2.1. The Timepix4 chip

The Timepix4 is a new chip developed by the Medipix4 collaboration aiming to improve upon both the Medipix3 and Timepix3. The chip is almost four times larger than its predecessor – 448 × 512 pixel matrix – while keeping the same pixel pitch – 55 µm. The design is optimized for the use of Through Silicon Via (TSV) technology, allowing for four-side buttability. This will significantly reduce the dead areas between modules when multi-modules approaches for large areas are used. To allow higher readout speeds, 16 high-speed gigabit wire transmitter (GWT) links are implemented in the chip, each of which can potentially output data at readout speeds up to 10.24 Gb s^−1^. When discussing frame rates and event rates for TEMPUS, we assume a more conservative speed of 5.12 Gb s^−1^ per link. As in Medipix3, the Timepix4 chip can be operated in a photon-counting mode. Hits are counted when input signal pulses in each pixel are higher than a predetermined threshold level. In this mode, the total number of hits per pixel during a frame is recorded in either 8- or 16-bit deep counters. Frame rates of up to 40 kfps in a continuous read–write mode (CRW) can be achieved. Much like Timepix3, Timepix4 can be also operated in a time-stamping – or event-based – mode. In this case, two parameters are registered per hit: every time the input signal goes above the threshold level, a time-stamp with 200 ps binning is recorded at the rising edge of the pulse – this is the time-of-arrival (ToA); in addition, the pulse length or time-over-threshold (ToT) provides coarse energy information with around 1 keV resolution. A data packet with this information plus the pixel address is sent immediately out of the chip for further processing. For moderate or very low photon fluxes as encountered, for example, in NRS applications, the time-stamping mode could also help to decrease the large data volumes compared with other detector systems. In addition, the chip can also time-stamp externally supplied signals, to allow time measurement relative to some external event. This signal, denoted as digital pixel, is thereafter registered directly as an event in a customized number of pixels in the pixel matrix. The fact that this feature is integrated into the chip itself makes the readout design much simpler compared with Timepix3-based systems, where the readout system had to provide it.

### 2.2. The chip carrier board

Acting as a physical support for the chip, and to connect it to the outside world, a single-chip board was designed at DESY. It uses several double-stage voltage translators to convert the 12 V input to a very stable 1.25 V needed for the chip’s operation. Although the form factor was not a strong requirement, the chip was placed at one edge of the carrier board to allow for more practical use in future experiments, for example to make it possible to surround a sample on up to four sides with multiple systems. The choice of material, Megtron6, as well as the electrical routing has been carefully made to allow the chip’s operation at its full performance. In particular, special care was taken to ensure that the high-speed traces from the Timepix4 GWT links to the readout board would have good signal integrity at 5.12 Gb s^−1^. Connectors and routing of other signals, such as the sensor bias voltage, and digital pixel used for time referencing can be found on the board as well. An array of thermal vias has been placed in the area directly below the chip to increase the heat exchange.

### 2.3. The readout board

As mentioned earlier, this first prototype was developed using a commercial Xilinx SoC evaluation board as a readout board. Specifically, the HTG-Z922 Xilinx ZYNQ^®^ UltraScale+™ MPSoC PCIe development platform was chosen (AMD, 2022 ▸). This SoC incorporates a CPU that can run a Linux operating system, and a field programmable gate array (FPGA) fabric. For high-speed IO, this particular device offers up to 32 GTH/GTY^®^ transceivers which can individually deal with data rates of 16.3 and 32.75 Gb s^−1^, respectively. During operation, the CPU is used to communicate with the control PC via a 1 gigabit ethernet (GbE) link using transmission control protocol (TCP). For testing purposes, a user can connect to the CPU on the Zynq directly using ssh. Python code running on the CPU is used for slow control of the Timepix4 chip, such as configuring the chip based on configuration files stored in the SoC. To do this, the CPU interfaces with the FPGA fabric, which is connected to the chip’s IOs. As well as interfacing with the chip, the FPGA executes fast control sequences, such as starting an acquisition in response to an external trigger signal. The high-speed readout is implemented using the SoC’s GTH transceivers; 16 of these receive data from the Timepix4 chip’s GWT links, and then, after data aggregation in the FPGA, the data are sent to the DAQ PC over Firefly^®^ optic fibers with 100 GbE, using user datagram protocol (UDP) as a protocol. In the future, the FPGA fabric could potentially also be used to directly implement data correction, calibration and reduction algorithms in the device, among other functionalities.

### 2.4. Mechanics

There are three major criteria concerning the prototype’s housing. Firstly, it should provide protection as well as mechanical stability to the different boards mentioned above. Secondly, it should supply an easy way to be mounted to different experimental set-ups. The third main criterion is the ability to cool all electronics components. To transfer the heat generated by the different elements, several fans have been installed in the housing to increase air circulation. It seems that temperature stable operation helps to improve the GWT’s stability. For reference, Timepix4 has a comparable power consumption to Medipix3, of around 1 W cm^−2^.

### 2.5. The data acquisition system (DAQ)

At the other end of the high-speed Firefly optic fibers, we have placed the Mellanox MCX516A-CDAT Connectx-5 Ethernet card. It consists of a 100 GbE dual-port QSFP28 directly connected to the DAQ server PC with PCIe4.0. Currently during operation the data are typically written directly to disk after beings received, and then processed offline. One of the main challenges ahead for this project is how to deal with the potentially very high data rates produced by the chip. A single chip working at a fast frame rate could already produce up to 80 Gb s^−1^ of data. These numbers will linearly scale up when multi-chip systems are produced to cover large active areas. It is foreseen that an accelerator card with built-in network links – such as a Xilinx Alveo FPGA card or Nvidia A100X GPU card – could be used instead in the future for further data processing (including data reduction) prior to saving to disk. High-speed data acquisition using accelerator cards has been demonstrated for other detectors; see for example Leonarski *et al.* (2023 ▸).

### 2.6. Control and data processing software

As mentioned above, Python code running on the CPU in the SoC provides various functions that control the Timepix4 chip and the detector as a whole – for example, configuring the detector and starting data taking. In turn, higher-level control software can call on these functions to carry out more sophisticated acquisition sequences. Currently, this also generally consists of running Python scripts directly on the SoC, by connecting to it via ssh. In the future, a gRPC interface will be implemented to allow beamline control systems and other software to issue commands to the detector. Data reception on the PC is currently performed using Python code to receive data packets from the 100 GbE link and save them to disk; the raw data stream from the detector can then be converted into meaningful data offline.

Some work has been carried out at DESY to read out Timepix3-based systems in the past. In particular, the Controlled Molecule Imaging (CFEL/CMI) group has developed a control, visualization and acquisition software called *Pymepix* to run commercially available Timepix3 devices (Al-Refaie *et al.*, 2019 ▸). Within this context, work has already started on a next version of this software to be fully compatible with the new Timepix4 chip. At this point in time, this package already allows us to have a real time online visualization of the data generated by TEMPUS. It is also foreseen for this software to eventually fully control the detector system. Some or all of this software may be upgraded to a faster language like C++ to improve throughput; *Pymepix* is structured so that performance-critical code blocks can be replaced without having to rewrite the entire software in the new language.

Additionally, software has been developed to calibrate and configure new Timepix4 chips before use. Firstly, the Timepix4 chip contains a set of digital-to-analog converters (DACs) which provide various reference voltages and currents; these settings need to be fine-tuned depending on factors such as the operation mode, incoming flux, *etc*. Also, each pixel has adjustable circuitry that can compensate for pixel-to-pixel variations in threshold to improve the image uniformity, and a threshold equalization is performed in order to find the optimal setting for each pixel (Rinkel *et al.*, 2015 ▸). This algorithm also leads to the creation of pixel masks where hot or noisy pixels are electronically masked so they do not produce spurious data packets.

## 3. Testing the system

The first tests in the lab were performed with a very restricted data bandwidth. Instabilities in the GWTs on the chip side (likely temperature related), plus issues with the required firmware on the FPGA side, were the main reasons for this. In particular, a debugging feature of the chip was used to perform the readout through the slow control link that is normally used to configure and control the chip. The hit rate capability of the system was therefore limited to roughly 5000 hits per second. This was however enough for testing basic functionalities, calibration and pixel equalization. Regardless of this limitation in the event rate, the system could also be used in experiments where the photon flux is low and high time resolution is a requirement. Therefore, we aimed for users/beamlines which may be interested in the time-stamping mode to have some background in fast (*e.g.* nanosecond) time-resolved experiments and the necessary infrastructure. The two NRS beamlines at PETRA III and at ESRF – P01 and ID14, respectively – fit those demands. Further, they provide so-called high-resolution monochromators (HRMs) with meV energy resolution at 14.4 keV, which allow for a very clean X-ray beam without any harmonics. These tests helped to speed up the development: from the fine tuning of the control software and the preliminary data analysis tools, to the improvement of our understanding of the chip and other components of the system.

### 3.1. Time resolution estimation

A TEMPUS detector using a 300 µm-thick p-on-n silicon sensor produced by Advafab OY (https://advafab.com/) was tested at the PETRA III P01 beamline. When detecting X-ray photons, the main factor limiting the time resolution is that photons can be absorbed at varying depths in the sensor, resulting in varying drift times to the pixel contacts. This effect will depend on photon energy; at higher photon energies the longer absorption length will lead to greater variation in the absorption depth. To achieve the best time resolution, ideally an electron-collecting sensor design should be used, to take advantage of the much higher mobility of the electrons than holes, and the sensor should be operated at a high voltage. Unfortunately, only a hole-collecting sensor was available. Moreover, high leakage currents were seen when biasing this particular sensor. So the voltage was limited to around 100 V.

Under these sub-optimal conditions, we can estimate the drift time of the charges (holes in our case) as follows: the hole mobility in silicon is μ_h_ = 450 cm^2^ V^−1^ s^−1^ (compared with μ_e_ = 1400 cm^2^ V^−1^ s^−1^ for electrons). We make the approximation that the field is uniform,
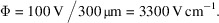
Note that using a higher bias voltage would linearly increase the field. We can then calculate the hole velocity using the mobility and the field,
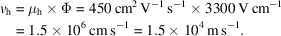
The drift time for 300 µm Si, 100 V bias and hole collection would then be
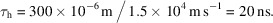


Only a few months after the initial tests at P01, the system was tested at the nuclear resonance beamline ID14 at ESRF. In this case a single high-speed data link running at 1.28 Gb s^−1^ was used which pushed the maximum event-rate to around 2 × 10^7^ hits per second. Also, a similar 300 µm-thick p-on-n sensor was used, but this time the leakage current was much lower. This allowed a higher biasing (up to 200 V), which increased the carrier drift velocity and thus improved the time resolution down to a few nanoseconds. Results of this experiment are presented in Section 3.3.

### 3.2. Tests at the P01 nuclear resonance beamline at PETRA III

The P01 beamline at PETRA III (Wille *et al.*, 2010 ▸) is dedicated to NRS and inelastic X-ray scattering [IXS and resonant-IXS (RIXS)] experiments at photon energies between 2.5 keV and 90 keV. The beamline offers high-energy-resolution monochromators in the meV regime. The maximum beam size is of the order of 1 mm × 1 mm. For the measurements, instead of impinging this beam directly on the much bigger sensor, we used the X-rays which were scattered from various target samples under 90° in a horizontal scattering geometry. This allowed us to illuminate the entire surface of the detector more homogeneously. Although we used several metals as targets to record elastically scattered photons and characteristic fluorescence radiation, particular focus was put on iron, having in mind further studies on the nuclear transition of ^57^Fe performed with the TEMPUS detector. For this purpose, the photon energy was set to 14.4 keV. The SR was running the so-called ‘timing mode’ during the tests. This mode consists of a total of 40 electron bunches per revolution. A full revolution takes 7.685 µs and the bunches are equally spaced at around 192 ns.

**Figure 3:**
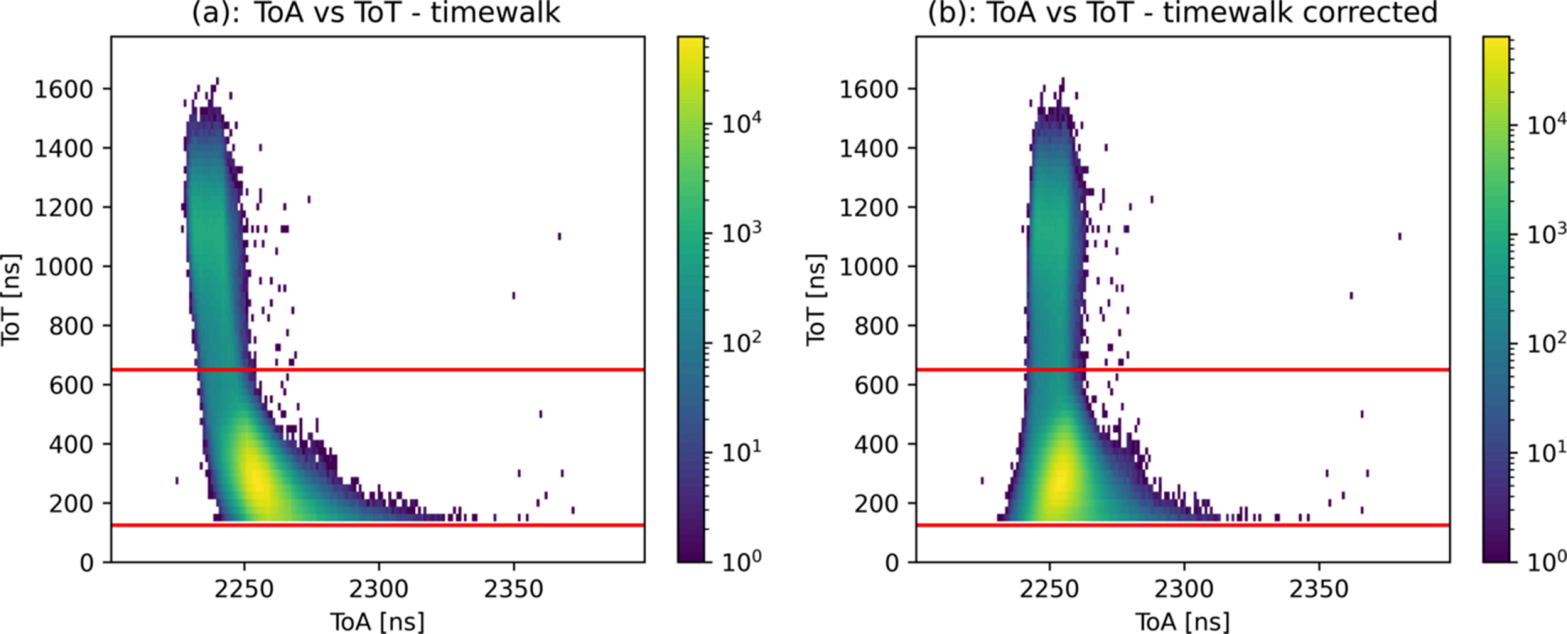
(*a*) ToT versus ToA plot showing the phenomenon known as time-walk. Signals with lower amplitude are registered at a later ToA due to their later crossing of the threshold. (*b*) The ToT versus ToA correlation is shown after the time-walk correction is applied. Lower ToT hits are now registered at the same time as the higher ones.

Note that the beam energy was set about 200 meV below resonance to ensure that no nuclear level excitation was produced. The detector was placed at a distance of approximately 5 cm from the target. In this configuration, elastically scattered photons, which have the same energy as the primary beam, and fluorescence photons, which are at a lower energy that depends on the target element, can be observed. Timepix4 can simultaneously measure energy and time information from individual events. Under certain conditions, this enables the separation of the different photon contributions (*e.g.* background, scattering, fluorescence) arriving at the detector. In the retrieval of the timing information, the so-called time-walk effect plays a role for low-amplitude signals: simultaneous signal pulses of different amplitude discriminated by a constant threshold are measured at different times. For the low-amplitude signals it takes significantly longer to reach the threshold than for larger ones. In consequence, the correlation of ToT and ToA for simultaneous events reflects the time-walk effect as shown in Fig. 3 ▸(*a*). As a result a function can be fitted to the correlation and for each event a ToA correction can be derived from its ToT measurement. The outcome of the correction is displayed in Fig. 3 ▸(*b*). The correction improved the time resolution values by an average of 3% for the high-energy distribution and 14% for the low-energy one. The final results after correction are presented later in this section and in Figs. 5 and 6. Note that for events with very low ToT, already close to the threshold value, the implemented time-walk correction shifts the ToA towards lower values. In the extreme case, some of them receive corrected ToA values lower than the prompt photons. This is an artifact of the time-walk correction itself which is made to improve the time resolution only. This effect has already been studied for Timepix3 (Yousef *et al.*, 2017 ▸).

**Figure 4:**
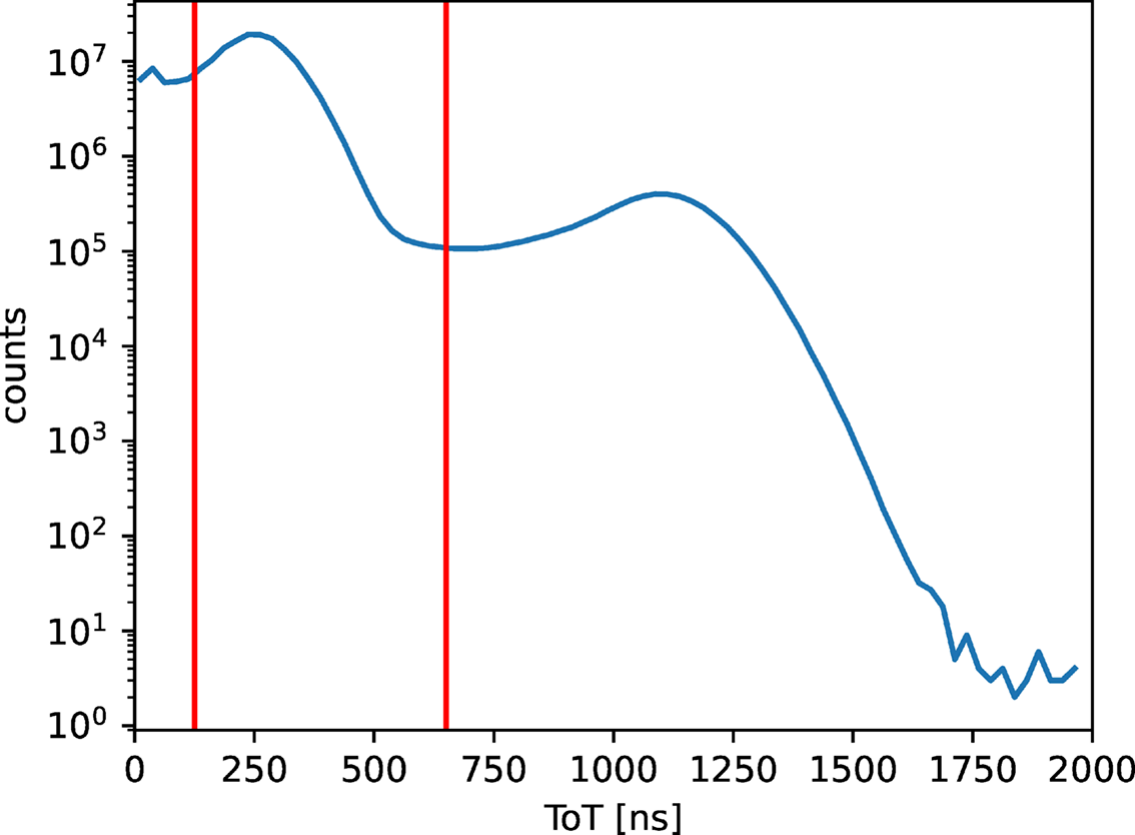
ToT spectrum. After setting a threshold at 100 ns ToT, two contributions are visible: the first one at around 300 ns ToT, which we attribute mainly to the 6.4 keV *K*-α fluorescence energy of Fe, and a second one at around 1100 ns ToT, which we attribute to the scattered photons at 14.4 keV. We set an arbitrary cut-off energy at 650 ns ToT and split all events into these two distributions.

The distinction between different photon energies can be demonstrated by studying the ToT distribution of the data shown in Fig. 4 ▸. For single photon events the signal amplitude and, as a consequence, ToT scale with the photon energy. After setting a threshold at 100 ns ToT, the first maximum is found at around 300 ns ToT, which we attribute mainly to the 6.4 keV *K*-α fluorescence energy of iron. We found the second maximum at around 1100 ns ToT, which we attribute to the elastically scattered photons. Note that, in order to improve the energy resolution (limited in principle by the chip to 1 keV), a full per pixel energy calibration should be used. Also, clustering algorithms are usually applied to bring together single events spread onto several pixels. These analysis methods, however, exceed the scope of the current work. We set an arbitrary cut-off energy at 650 ns ToT and split all events into these two distributions: low and high energies. Note that a long tail of the lower energy distribution is likely due to hits just above the threshold where fluctuations in electronic noise will have large and asymmetrical effects on when the hit is recorded. A higher cutoff could improve this at the expense of reducing the signal.

**Figure 5:**
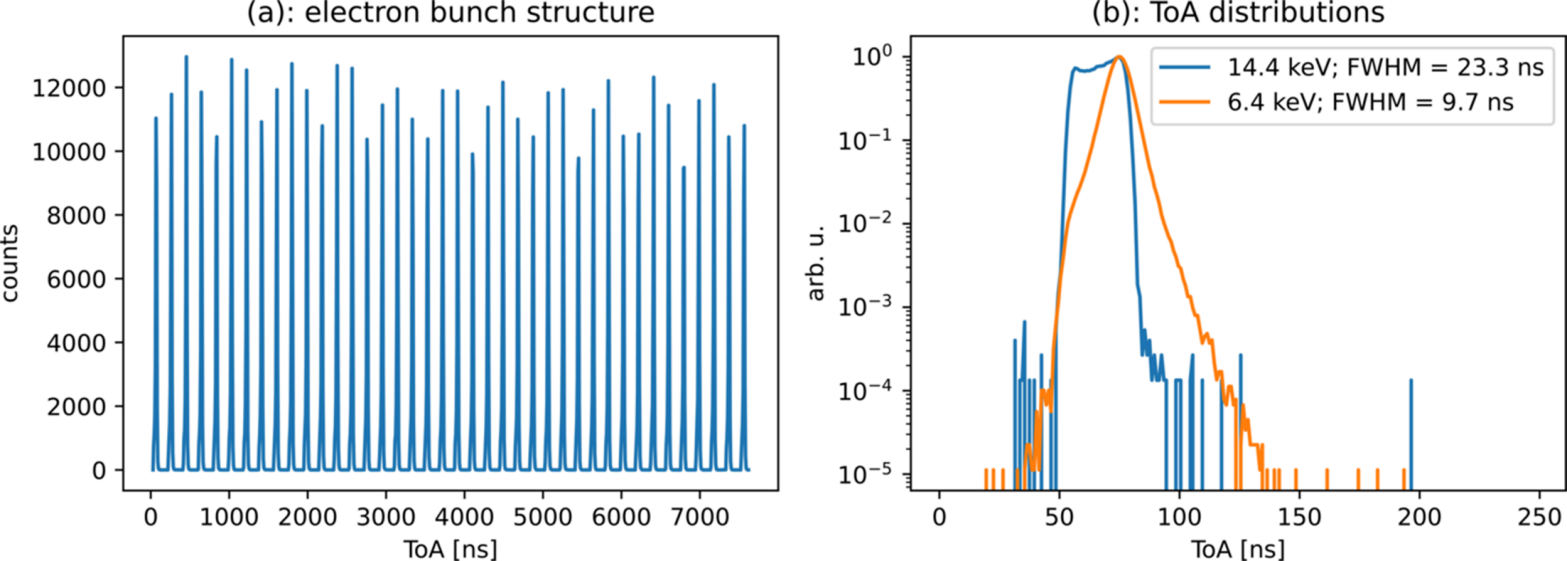
(*a*) Events binned by ToA obtained for several revolutions of PETRA III (initialized once per revolution). Hits corresponding to the 40 different electron bunches can be clearly distinguished due to the high time resolution of TEMPUS. (*b*) ToA binning of the two different TOT contributions divided by an arbitrary threshold placed at 650 ns ToT are shown. The obtained time resolution, defined as the FWHM of the distribution, corresponds to the expected values. The higher time resolution of the lower energy photons is due to the shorter absorption length in silicon. As mentioned in the text, time resolution was limited by the low biased silicon sensor: 23.3 and 9.7 ns, respectively.

We used this information to bin the events by ToA. This can be seen in Fig. 5 ▸(*a*). Using an attenuated flux, we identify photons coming from each individual electron bunch produced by PETRA III. Results show the ToA of individual events with respect to the beginning of each revolution. Due to the already mentioned limitations on hit rate set by the readout system, the experiments had a relatively low duty cycle, where hits were recorded over a short time period (between 80 and 1600 µs) followed by a longer pause for readout. Therefore, data from many revolutions were combined to attain sufficient statistics. The individual bunches of the PETRA III machine operating in the timing mode are clearly visible. As mentioned above, the bunches are equally spaced 192 ns apart. Note that, for a detector working in a frame-based mode, the required frame rate to achieve this result must be at least 5.2 MHz with the subsequent large volume of data produced. Due to precise timing references by the digital pixel inputs, the ToA of the events can be aligned to the timing of the bunch clock instead of the revolution clock. The so-produced ToA distribution is shown in Fig. 5 ▸(*b*). The time resolution, in this case defined as the full width at half-maximum (FWHM), of 23.3 ns for the high- and 9.7 ns for the low-energy photons is in good agreement with the expected value of 20 ns for the high-energy photons and the relatively low bias voltage used across the sensor. The very different shapes of the two distributions are also expected. The non-flat and asymmetric top of the higher energy distribution is explained by photons converted very close to the entrance window of the sensor. The carriers produced there will take around 20 ns to arrive at the other end of the sensor. As for the photons converted deeper in the sensor, the travel time of the carrier is shorter, which implies a lower ToA, but due to the attenuation produced by the silicon sensor itself their number will be smaller. The better time resolution obtained for low-energy photons is explained by the fact that they are all absorbed in a very shallow thickness of the sensor.

### 3.3. Tests at the nuclear resonance beamline ID14 at ESRF

The new nuclear resonance beamline ID14 at ESRF is also dedicated to NRS experiments. Here too, several metals were used as target and again emphasis was put on iron. The photon energy was set to 14.4 keV and the SR was running the 7/8 + 1 filling mode during the tests (ESRF Filling Modes; https://www.esrf.eu/Accelerators/Operation/Modes): 7/8 of the ring is filled with 868 uniform bunches and the remaining 1/8 is filled in its center with a relatively large single bunch. As mentioned above, a new assembly was used with a sensor that did not show any leakage current issues at higher voltage.

**Figure 6:**
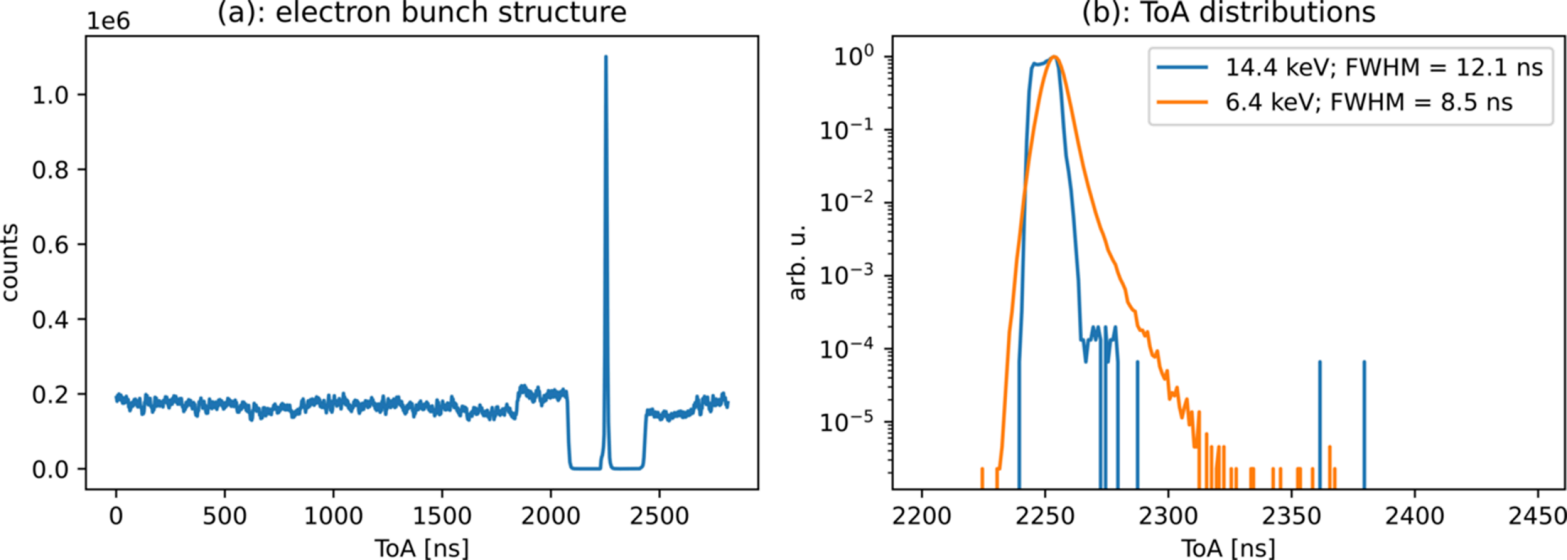
(*a*) Events binned by ToA obtained for several revolutions of ESRF (initialized once per revolution). The 7/8 + 1 electron bunch structure is visible. (*b*) The low- and high-energy contributions are shown as a function of ToA. Due to the higher bias voltage, an improve time resolution was achieved: 12.1 ns and 8.5 ns, respectively.

After performing the time-walk correction and energy discrimination that were mentioned above, and shown in Fig. 3 ▸, the ToA distribution obtained for the whole revolution is also shown in Fig. 6 ▸(*a*). The structure of the electron bunches in this mode with the single bunch in the middle is clearly visible. The ToA distribution obtained for the single bunch seen above is also shown in Fig. 6 ▸(*b*). In this case, and since the bias voltage has been increased, the time resolution achieved for the two different photon energies are 12.1 and 8.5 ns, respectively. As mentioned earlier, the artifact on the time-walk correction wrongly assigned lower ToA values to events with low ToT values. That explains the fact that there seems to be events of the lower energy distribution arriving earlier than those from the higher energy distribution. Also, the 14.4 keV photons incorrectly attributed to the low-energy distribution, due to charge sharing, explain the long tails of the latter.

## 4. Conclusions and outlook

The single-chip TEMPUS prototype is being developed by DESY with the aim to replace LAMBDA detectors currently deployed at PETRA III. The use of a new, more performing chip – Timepix4 – will make working at a high frame rate possible. In addition, it features a time-stamping mode, which provides event-based X-ray detection with few-nanoseconds time resolution. The system has been described and the prototype has seen its first light. Further characterization and calibration tests are ongoing. Preliminary results from NRS beamlines at PETRA III and ESRF show that the achievable time resolution of a few nanoseconds for X-rays is currently limited by the sensor used, a 300 µm-thick hole-collecting silicon sensor. This resolution is sufficient to fully resolve the 40 electron bunches of the timing mode at PETRA III and the single-bunch in the 7/8 + 1 mode at ESRF. Improvements in this area could be achieved by using different types of sensors such as electron-collecting silicon sensors (achieving potentially three times faster drift times), or the use of low-gain avalanche detector technology (Pellegrini *et al.*, 2014 ▸) (potentially improving time resolution by boosting the signal). Alternatively, the spread in absorption depth can be reduced by using high-*Z* materials such as GaAs and CdTe. All these options are currently being considered. For other experiments, such as ion and photoelectron imaging, the use of visible-light sensors is foreseen and it could significantly improve the time resolution, bringing it closer to the limit of the Timepix4 chip itself, as has already been done with Timepix3-based detectors (Bromberger *et al.*, 2022 ▸).

Moreover, data acquisition through the newly developed high-speed data-links could be demonstrated. Ongoing development will enable the use of all 16 links, and achieve their maximum data rate of 5.12 Gb s^−1^ (or total of 80 Gb s^−1^). Further tests with the single-chip prototype in collaboration with users are foreseen. Besides the ongoing collaboration in the field of NRS, the XPCS community is interested in this development to access the fast dynamics in the sub-microsecond regime. Moreover, and in collaboration with other partners, a multi-chip module is also under development.
